# Producer perspectives on the constraints to aquaculture development in the US Great Lakes region

**DOI:** 10.1371/journal.pone.0340682

**Published:** 2026-01-29

**Authors:** Haley A. Hartenstine, J. Stuart Carlton

**Affiliations:** Department of Forestry & Natural Resources, Purdue University, West Lafayette, Indiana, United States of America; Mansoura University, EGYPT

## Abstract

Despite significant federal interest and the vast resource potential of the region, the land-based food fish aquaculture industry remains relatively stagnant in the U.S. Great Lakes states. In this study, we use the Theory of Planned Behavior to explore the factors influencing aquaculture producers’ intentions to expand or diversify their operations. We conducted semi-structured interviews with 34 food fish producers across the eight Great Lakes states. Our thematic analysis revealed that while most producers expressed positive intentions to grow, these intentions were often constrained by low perceived behavioral control. Major barriers included limited access to capital, regulatory complexity, inadequate institutional support, and challenges in public perception. Attitudes toward expansion were shaped by both mission-driven motivations, such as supporting local food systems, and pragmatic concerns about cost, risk, and labor. Subjective norms were overwhelmingly favorable, reflecting a strong sense of community and peer support within the industry. Past experiences with expansion further influenced current intentions, as well, reinforcing cautious, incremental growth strategies. These findings suggest that policy reforms and structural support, particularly in financing, regulation, and outreach, are critical to unlocking the growth potential of aquaculture in the Great Lakes region. By centering the voices of producers, this study provides actionable insight into the systemic barriers that must be addressed for meaningful industry advancement.

## Introduction

Aquaculture plays a vital role in meeting rising global demand for seafood, providing essential nutrients to billions of people and contributing significantly to food security, especially in low- and middle-income countries [1–3]. Aquaculture has emerged as a cornerstone of global food systems and, in the last decade, has begun supplying over half of the aquatic animal products consumed worldwide [4]. In 2022, global aquaculture production reached a record 130.9 million tonnes, accounting for 59 percent of total output of the fisheries and aquaculture sector and contributing 57 percent of aquatic animal foods destined for human consumption. This growth has been driven largely by countries in Asia, which combined produce over 90 percent of the world’s farmed aquatic animals, with China alone responsible for more than a third of global aquaculture output [1,5].

The United States has lagged in aquaculture production and natural fish stocks remain a finite resource that is insufficient to meet demand at an acceptable price [6,7]. As a result, seafood demand is largely met through imports: according to the National Marine Fisheries Service (NMFS), between 70–85 percent of the seafood consumed in the US is imported, and about 50 percent of this is produced through aquaculture. In 2019, the US seafood trade deficit had grown to $16.9 billion, a substantial increase from the $9.5 billion deficit reported just a few years prior, and is predicted to continue rising [8,9].

Although the U.S. is the world’s third most populous country [10], the U.S. aquaculture industry ranks 17th globally in production [8]. While global aquaculture has been one of the fastest-growing food production sectors for decades [5,11], U.S. growth has lagged, with some years showing negative production trends [12]. Yet the U.S. possesses significant untapped potential for aquaculture expansion, supported by extensive coastal and inland water resources. This is especially true in the Midwest: the twelve states of the USDA North-Central Region account for one-third of U.S. farms and nearly 40% of the market value of U.S. crops (figures calculated from [13] in [14]). Aquaculture in this region includes food-fish production of trout (generally rainbow trout, *Oncorhynchus mykiss*), yellow perch (*Perca flavescens*) and other salmonids (*Salmonidae*), catfish (*Ictalurus punctatus* and *I. punctatus* × *I. furcatus* hybrid), tilapia (*Oreochromis spp.*), and hybrid striped bass (*Morone saxatilis* × *Morone chrysops*), raised primarily in ponds, recirculating aquaculture systems (RAS), raceways, and, for shrimp, biofloc systems [15,16]. Despite this capacity, the USDA North-Central Region states account for less than 2%, and the broader Midwest less than 3%, of U.S. farmed food-fish sales—a figure stagnant or declining for over two decades [14,17]. Identifying barriers to growth in this high-potential region is critical to advancing national food security goals.

There has been increasing federal interest and investment in domestic aquaculture. In recent years, the US government has invested in aquaculture programs, grants, and research to support industry growth, including a 2020 presidential executive order encouraging growth of the industry [18]. To ensure these programs are effective and efficient, it is essential to understand barriers to growth of the industry, and therefore which areas to delegate more research, funding, and structural support.

Previous research has identified key barriers to aquaculture growth nationally, including restrictive regulations, prohibitive expenses, negative public perception (e.g., [19]), and ineffective policy mechanisms [20,21]. However, there is a need to examine these factors from the producers’ perspective and within the unique context of production in the US Great Lakes region. This study addresses this geographic and sectoral gap by investigating the factors that shape producers’ decisions to expand or diversify their businesses. This study applied the Theory of Planned Behavior [22] to address the research question of how attitudinal, normative, and behavioral control factors influence producers’ intention to expand or diversify land-based food fish aquaculture businesses in the US Great Lakes region.

## Methods

### Theoretical framework: The Theory of Planned Behavior

**Fig 1:**
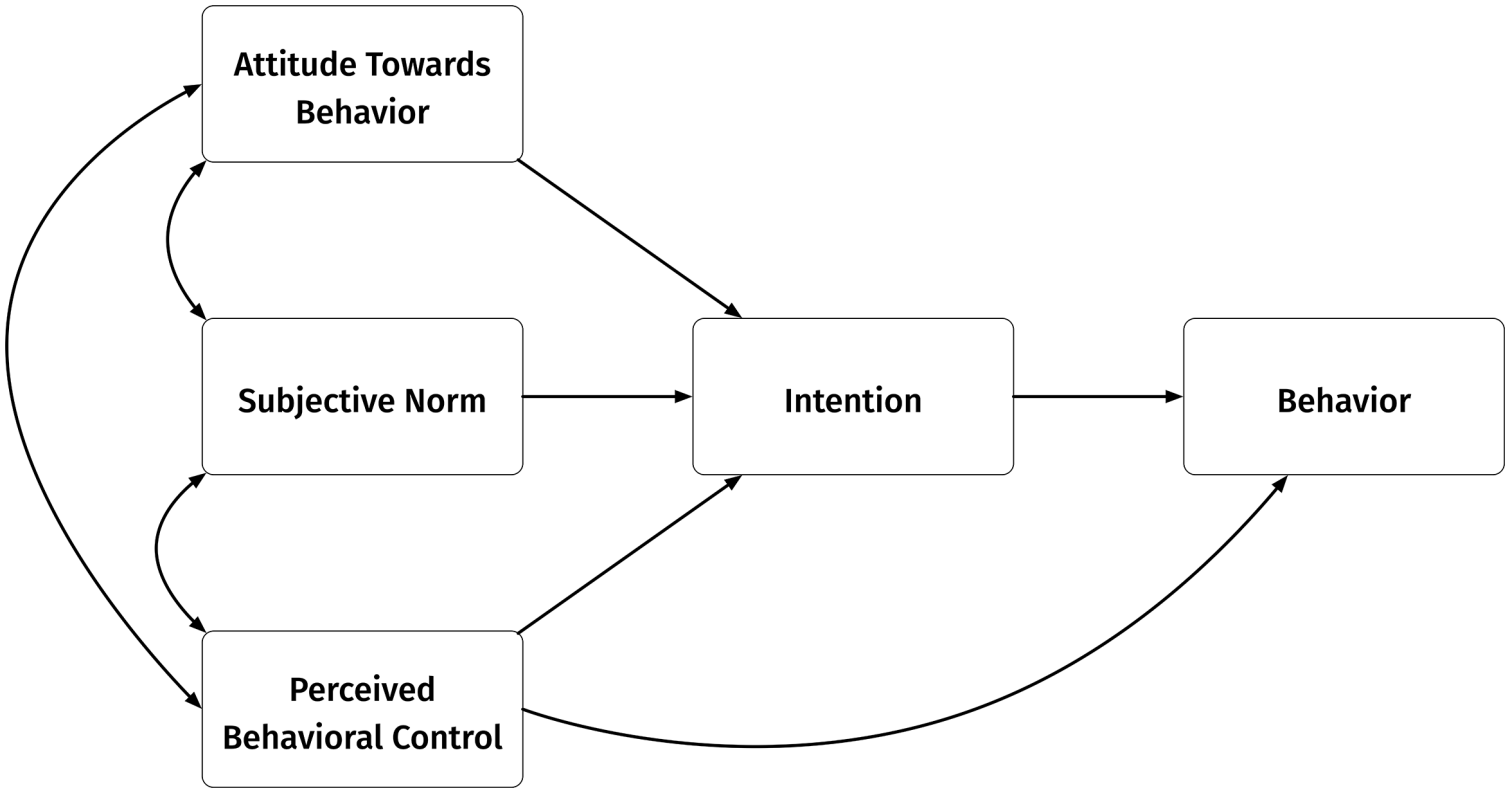
Simplified model of the Theory of Planned Behavior, adapted from Ajzen [23].

The Theory of Planned Behavior (TPB) explores influences on an individual’s intention to perform a behavior. This behavioral intention is informed by three core constructs: an individual’s positive or negative *attitude* toward the behavior; the perceived social pressure, or *subjective norm*, to perform it; and the perceived ease or difficulty of performing the behavior, known as *perceived behavioral control* (PBC; Fig 1). Building on studies that have successfully expanded the model for specific contexts (e.g., [24]), we also incorporate past behavior to enhance the framework’s analytic power in an industry where past successes and failures are particularly salient. The Theory of Planned Behavior (TPB) has been widely used across agricultural and natural resources science to understand farmer intentions to diversify their production [25], adopt sustainability best management practices [26,27], and implement modern pest management techniques [28], among other farm management decisions. For a more detailed discussion of TPB in this context, please consult Hartenstine [29].

In the Fall of 2020, we developed an interview guide using TPB as the underlying theoretical framework (see the full guide in [29]). We divided the guide into sections designed to address each of the three main theoretical constructs: attitude toward the behavior, subjective norms, and perceived behavioral control. We included past behavior as an additional construct and a section of questions regarding the actual intentions of the interviewees. Asking producers directly about their behavioral intentions regarding expansion or diversification of their farm allows us to work backwards through the theoretical framework of TPB to determine what constructs predominantly influence farmers’ behavioral intentions.

The interview guide was reviewed by experts in the field, edited, and pilot–tested with aquaculture producers outside of the region (i.e., who were not included in the study). Based on the pilot–testing feedback, we made minor adjustments to the questions to improve clarity. The guide was approved by the Purdue University Institutional Review Board, protocol IRB-2020–1842.

### Research paradigm and researcher characteristics

Given the fact that we are adapting existing theory to help explain a real-world phenomenon, this approach is broadly post-positivist, though we are also informed by the pragmatic reality that TPB is broadly applied to situations such as these. We also acknowledge that researcher characteristics can influence interpretation of qualitative research. The researchers in this study were a graduate student and a research faculty member who were familiar with, but not active participants in, the aquaculture industry. One of the authors (JSC) serves as a PI on the Great Lakes Aquaculture Collaborative, which is a National Oceanic and Atmospheric Administration/Sea Grant-funded project to improve aquaculture outreach in the Great Lakes region. The interpretation of the data is inherently informed by these, and other, researcher characteristics.

### Recruitment and interview process

We used purposive sampling to recruit food fish producers to participate through our professional networks. We set an initial goal of interviewing at least five producers in each of the eight Great Lakes states: Indiana, Illinois, Michigan, Minnesota, Ohio, Pennsylvania, New York, and Wisconsin. This initial target of five interviews per state was what we considered to be a reasonable goal, not a magic number. We chose this number while acknowledging that we may end up needing more or fewer interviews depending on the level of saturation that we see develop as the interviewing phase progresses. Saturation is reached when additional interviews are not producing any new emergent themes ([30]: p. 135). Although saturation was our biggest consideration when determining how many interviews we would ultimately conduct, we were also limited by the number of producers in each Great Lakes state that fit our criteria to be interviewed.

**Fig 2:**
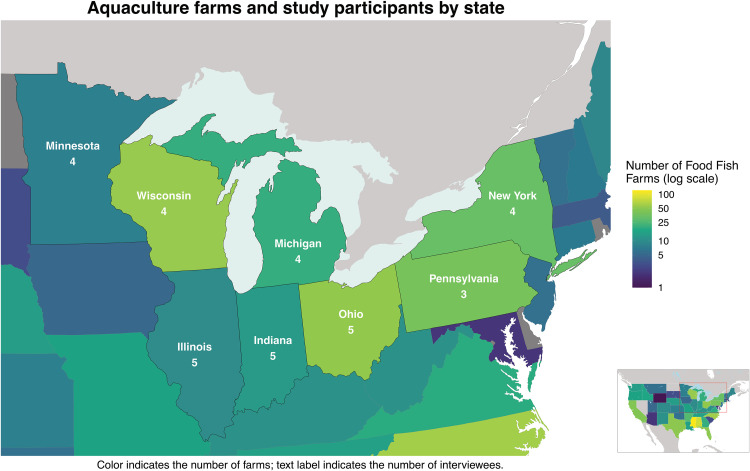
A descriptive map of the study area. Color fill indicates the number of food fish farms in that state (note the log scale; data from USDA 2024). White text indicates the number of interviews conducted in the state. Map boundaries drawn from U.S. Census Bureau and Natural Earth.

Ultimately, we interviewed 34 producers: 5 in Illinois, 5 in Indiana, 4 in Michigan, 4 in Minnesota, 4 in New York, 5 in Ohio, 3 in Pennsylvania, and 4 in Wisconsin (Fig 2). The redacted interview transcripts are available for review at the Purdue University Research Repository [31]. Given the relatively small size of the food fish industry, the extremely low number of food fish producers in certain Great Lakes states, and the fact that in some states we interviewed all known producers, we do not disclose detailed demographic information about the participants in this study in either the transcripts or in this manuscript. We also do not disclose the state in which a participant is located when discussing their responses. These decisions were made as a measure of protection for the confidentiality that was promised to the participants, some of whom could be too easily identified solely by demographic information or the inclusion of a statement they made accompanied by the state they are located in. This small sample size accurately reflects the modest size of the food fish aquaculture industry in the Great Lakes region.

We recruited people and conducted the interviews over Zoom between April 1 and November 30, 2021. Each interview lasted approximately 30–60 minutes. We obtained verbal consent to participate, which was documented by the interviewer (HAH). With the interviewee’s permission, we recorded and transcribed the audio, removing identifying information from the transcripts. After transcription, we entered the transcripts into NVivo for coding.

Our first step was to get familiar with the data. As we read through each transcript, we simultaneously drafted a codebook using a combination of deductive and inductive thematic coding. We began deductively, developing a broad outline of the codebook that included latent themes grounded in TPB. This outline served as a starting point, which we substantially expanded with semantic themes and subthemes that emerged inductively as we read through each transcript. Some of these emergent themes aligned with TPB constructs, while others did not.

To calculate intercoder reliability, three coders independently coded three interviews using the codebook. After completing the coding, we uploaded all nine transcripts (three from each coder) into NVivo and ran a coding comparison query. This query produced an average Cohen’s kappa coefficient of 0.81—a statistical measure that accounts for agreement occurring by chance. A kappa above 0.75 generally indicates substantial agreement between coders, and a kappa of 1 represents perfect agreement. Our average kappa of 0.81 suggests the coding is reliable and supports the validity of our analysis [32]. After discussing and refining the codebook to address discrepancies, we completed the coding for all 34 interview transcripts.

## Results

We present findings for each construct of the Theory of Planned Behavior (TPB) by showing the prevalence of themes with frequency data and revealing depth with illustrative quotes from the interviewees.

### Behavioral intention

Behavioral intention, which is the primary outcome in the TPB framework, was defined as a producer’s intent to expand or diversify their business. Of the 34 participants, a majority of 26 (76%) expressed a positive intention to do so, while 8 (24%) did not (Table 1).

**Table 1:** Behavioral intention themes and example quotes.

Behavioral Intention		
Theme	# of interviews	Exemplary Quotes
**Positive**		
Will or might expand/diversify in future	26 (76%)	“Yes. I intend on expanding every single year.”
**Negative**		
Will not expand/diversify in future	8 (24%)	“Why would I want to? You know, I’m making enough money, I’m comfortable, you know, I’m happy. It’s a wonderful life. Why would I want to make my life more complicated?”
**Types of future expansion**		
Physical expansion	22 (65%)	“...we had six tanks of fish, we’re adding six more tanks of fish, but we’re taking away grow space from plants. So we’re expanding the fish part of it, but reducing the plant part of it.”
Market diversification	13 (38%)	“As far as future expansions, additions...future markets, that would be, you know, a bigger customer base, that is definitely something we’re going to have to be looking at.”
Species diversification	11 (32%)	“Right now actually just rainbow trout and we’re hopefully looking to expand to add some other fish to our farm, but right now just rainbow trout.”
Expansion of fish yields	8 (24%)	“I don’t think I’m going to go out and build more raceways. But if we could tweak our numbers and cycle them a little bit different...maybe expansion is just changing your feed, to get better growth. Maybe that leads to 20,000 pounds more at the end of the year, just because you change to a better diet. So yeah, anything I can do to produce as many pounds as I can, without physically physical features.”
System diversification	5 (15%)	“So we would probably take one pond, try and switch it to [RAS]. We’re talking about doing an indoor system.”

#### Negative behavioral intention.

Analysis of the eight producers (24%) who did not intend to expand revealed two distinct mindsets: those content with their current operational scale and those who felt constrained from growing. The constraints cited were varied. For some, it was a matter of age and health, with one participant noting they were at a point where they “should be retired” and not “reinventing another job.” For others, regulatory burdens were a key barrier to growth: “I’m happy with how it is now. Only because I don’t want to deal with the regulations no more”.

Recent economic shocks, most notably the COVID-19 pandemic, also played a role, with some producers needing to focus on immediate recovery before contemplating expansion:

“Right now, we’re happy with the way it is. We have to recover from the loss of business that we suffered over the last year and get things back up again and getting all our tanks full of fish. So no, no plans immediately to add on.”

However, it is unclear whether these individuals would plan to expand or diversify once they have recovered from the losses of the pandemic and regained a sense of normalcy in their business operations. Given the right conditions, some expressed willingness and open-mindedness about expansion and/or diversification of their farms.

#### Positive behavioral intention.

Among the 26 producers (76%) expressing positive behavioral intention, commitment levels ranged from vague future aspirations to projects already in progress. Physical expansion was the most common intention, with 22 of the 34 total participants (65%) either considering, planning, or actively constructing additions such as new raceways, ponds, tanks, or facilities (Table 1). Producers also intended to diversify their businesses by pursuing new market channels, growing different species, or incorporating new production systems like recirculating aquaculture systems (RAS). This intention was often linked to a belief that adaptation is essential for survival. As one producer put it:

“(Y)ou probably need to be changing or growing. Because you either grow and change or you go the opposite direction. And eventually then you’re, you don’t exist. So, I’d say you need to be profitable. And you need to be growing and changing with the times.”

### Attitude toward the behavior

Attitudes toward the behavior reflect a producer’s favorable or unfavorable evaluation of expansion or diversification. Nearly all interviewees expressed a mixture of both, acknowledging the significant benefits as well as the inherent difficulties of growth (Table 2).

**Table 2:** Attitudes toward the behavior themes and example quotes.

Attitudes toward the behavior		
Theme	# of interviews	Exemplary Quotes
**Favorable**		
Environmental Sustainability/ Local	25 (74%)	“...I just see it being an opportunity to do sustainable farming and feed a lot of people.”
Success = profitability	23 (68%)	“...when it gets right down to it, you got to raise a product profitably. If you’re not making money out of it, it’s not going to last...you got tobe able to turn a profit and produce something that people will come back for.”
Meets consumer demand	17 (50%)	“Oh, well, I already plan on [expanding]. I just look at the size of the market, the huge demand for fish...”
Rewarding industry	5 (15%)	“...being able to care for the animals, but then also producing something that improves peoples lives that they enjoy...you know, anything aquaculture related is pretty fulfilling for me.”
Family business	5 (15%)	“...I’d love to build the farm up to where it’s a very nice, profitable thing, maybe pass it down to, you know, my kids...”
**Unfavorable**		
Lack of support	19 (56%)	“I don’t think they care about it. It’s so minute that they don’t even feel it’s it’s worth even in the colleges to even have any kind of an aquaculture program.”
Expensive industry	16 (47%)	“It’d be an extremely expensive profession to just start from scratch I would think. You pretty well would have to have money I would imagine to get you know, to get into it.”
Low profit margins	15 (44%)	“...it’s a low, food fish is a very low margin industry. So when it comes to margins in the fish industry, it’s, you know, bait fish at the top, stocking fish in the middle, and then really bottom is food fish.”
High risk industry	13 (38%)	“No, because there’s a lot of risk involved. And a lot of people are not going to want to take the kind of risks that we take, or they’re not going to have the financial resources at risk that they want to put at risk.”
Excessive workload	13 (38%)	“For a lot of people, I would imagine the work is a deterrent, aquaculture is a tremendous amount of handwork.”
Lack of knowledge	10 (29%)	“It doesn’t hurt to have a good biological, aquatic biology background. That’s a real problem for a lot of these folks, they think it’s easy money and it’s easy...and aquaculture is agriculture. Most, you know, farmers don’t come in blind...”
Expanding = more regulation	6 (18%)	“We’re limited by the number of fish we can raise...I can only raise 20,000. I’m not going to raise 22,000 lbs I’m gonna only raise 20,000 pounds which puts me under the 20,000 lbs before I have to start filling out all these NPDS permits.”
Environmentally Unsustainable	3 (9%)	“Right now, if anybody tells you it’s truly sustainable, that’s not true. You know, we still use fish meal, we still kill kill fish to feed fish.”

#### Profitability.

Profitability was a primary factor influencing attitudes toward expansion. Producers noted that while necessary for business survival, the potential for profit varied greatly by market. A recurring theme was the hesitancy to expand in the food fish market due to its low margins compared to stocking or bait fish. As one producer who serves multiple markets explained:

“...food fish is a very low margin industry. So, when it comes to margins in the fish industry, it’s, you know, bait fish at the top, stocking fish in the middle, and then really bottom is food fish. I don’t know why it’s higher margin to feed other fish for sport than it is to feed our own people. But that’s how things are.”

These low margins were exacerbated by competition from cheaper imported seafood, making it difficult to justify local production without a high-value species. As one respondent stated, “...it’s cheaper to import than it is for people to justify buying something grown closer to home. And I can’t fix that.”

This attitude extended to diversification through processing. Producers viewed processing unfavorably, expecting it to narrow their margins even further. One participant stated plainly, “Yeah, most of us have been avoiding [processing]. Just because we don’t want that responsibility, and it just lowers the amount of money you can get for the fish, they’re worth more alive than they are processed.”

#### Expensive, labor intensive, and risky.

The consumer’s willingness to pay is not the only underlying issue that producers blamed for low profit margins of food fish. Many of our interviewees agreed that aquaculture as an industry is extraordinarily expensive and involves high levels of risk. These two factors varied depending on the producer’s type of production system (i.e., outdoor ponds were less expensive than indoor RAS but posed more risks such as predation), but the capital-intensive nature of the business was a common unfavorable attitude. One participant described the cascading financial and operational risks as follows:

“And if you want to do something indoors, then you get into the financial side of it, where you’re going to have to have big vats, you’re gonna have to have enclosure and heating, you’re going to have oxygenators, you’re going to have pumps. Now, all of the sudden, you’re getting into a lot more financial, and then it’s a little scary in itself. Because you know, something goes down, you got to make sure you got generator backups, because you could instantly lose oxygen, your generators or your power goes out for two hours, you could lose all your fish. So, there’s a high risk there.”

Similarly, the heavy workload required for expansion was a significant deterrent for 13 of the 34 participants (38%). Many felt they were already at capacity and did not wish to take on more work, a sentiment captured by one producer:

“So, it’s pretty much just the fish and honestly, that’s about all we can handle because when we are busy—it’s nice to think that we could be doing something else, we’ve got the property to do it. It’s just the manpower and the time is the problem.”

#### Environmental sustainability and local foods movement.

Counterbalancing these concerns were highly favorable attitudes rooted in pride and market opportunity. Producers felt aquaculture was a rewarding industry that provides a high-quality product to local consumers. Many perceived a growing demand for domestic seafood, a trend that they felt was amplified by the COVID-19 pandemic’s exposure of fragile long-distance supply chains:

“Having the food source closer to the market, I think is a huge, I mean, obviously, you know, going through COVID people, I think, started to realize that a little bit that those long-term long-distance food chains are not the best idea.”

This enthusiasm for local food was often accompanied by a commitment to environmental sustainability. A widespread belief was that land-based aquaculture contributes positively to both movements and will become more critical as traditional fisheries face pressure from pollution and climate change. One producer saw a clear need for “more controlled cultivation of species…to meet the food demand.” In contrast to this view, only 3 of the 34 participants (9%) voiced a belief that aquaculture is not environmentally sustainable.

### Subjective norms

Subjective norms, the perceived social pressure to engage or not engage in a behavior, were the most straightforward and least nuanced constructs in this study. The findings were overwhelmingly positive in valence: 31 of 34 participants (91%) expressed strong peer support for expansion and diversification, while only 3 (9%) felt a lack of support or competitiveness.

#### Peer support.

The primary theme explaining these favorable norms was a shared belief that the aquaculture industry is not yet saturated or overly competitive. Producers described a sense of “spaciousness,” believing there is enough room for everyone to succeed. As one participant noted:

“In the Midwest Great Lakes region, we’re still for the most part a niche market. I mean, we’re not a commodity where it gets cutthroat, plenty room for everybody. And oh, yeah, I’m supportive of other people expanding as long as they got a good business plan, and they got their head on thinking right.”

This perspective fostered a sense of collegiality and a “rising tide lifts all boats” mentality. Producers felt that individual success benefits the entire industry by strengthening the community and creating more opportunities to learn from and support one another. One interviewee expressed this shared sentiment:

“I think, for the most part, everybody sees you know, success, as generally positive… success for anybody as being generally positive. You know, rising tide lifts all boats. And I think that’s the way most producers see it. We don’t find ourselves in direct competition a lot. And so there’s a pretty high level of collegiality and cooperation.”

This belief was reinforced by a mutual respect born from shared struggles. Participants recognized how difficult the industry can be and described a strong sense of community where producers help each other, knowing it strengthens the industry. Our interview results suggest that subjective norms are collectively favorable amongst aquaculturists regarding expansion and diversification. The key themes we identified in the interviews regarding subjective norms included high levels of peer support, respect, and community amongst producers. Collectively favorable subjective norms contribute to positive behavioral intention, and in our case do not pose a barrier to expansion or diversification.

### Perceived behavioral control

Perceived behavioral control (PBC), defined as a producer’s perceived ease or difficulty of expanding or diversifying, was the most complex construct in our study. While responses varied, several key themes emerged that reveal significant barriers to the growth of aquaculture. All the major themes can be seen in Table 3. We focus here on five major themes: access to knowledge, financial resources, regulations, support systems, and social license.

**Table 3:** Perceived Behavioral Control themes and example quotes.

Perceived Behavioral Control		
Theme	# of interviews	Exemplary Quotes
**Easy**		
Access to knowledge	26 (76%)	“But information is readily available. I think if you if you want to, if you know where to look, and you know, what to look for.”
Fair regulations	24 (71%)	“I don’t think regulations are a hindrance at all...because there aren’t a whole lot of regulations in the aquaculture industry right now, to be honest with you, at least, from what we’ve gone up against.”
Markets/Marketing	14 (41%)	“Absolutely, they would not believe the amount of fish that we’re selling locally in our hometown, which is booming right now. As far as the restaurant fish go, I can’t even believe it myself.”
Access to natural resources	10 (29%)	“But given our resources, here, we sit on an aquifer and just looking for something to do when I did come across aquaculture and started studying, it just seemed like a real good fit.”
Access to financial resources	7 (21%)	“...because we’ve been fortunate enough, up to this point that we haven’t had to seek out any outside capital or investment, it’s all been done with, with our own personal investment.”
Access to processing	7 (21%)	“Yep, we have our own processing facility on the farm...so we grow them at the farm and process on the same site.”
Access to labor	6 (18%)	“...as far as managing water and moving water and filtering water and aerating water, and those sort of things we’ve had absolute wonderful access to labor, in the sense of the builders and the doers of running it on a daily basis.”
**Hard**		
Lack of access to financial resources	26 (75%)	“Capital is on everyone’s mind. Spending money is risky. For sure, the number one reason people don’t build bigger has to do with access to capital. And the confidence from capital lenders in aquaculture industry is they consider it very risky and low profit.”
Social license	17 (50%)	“...it’s not just consumer perception, it’s societal perception, and a lack of social licence for the industry to be able to operate efficiently and to grow. And that’s why we don’t see growth in the US aquaculture industry.”
Over-regulated	13 (38%)	“They keep us from expanding. Regulations keep aquaculture behind. I mean, the USA is so far behind compared to the world it’s embarrassing.”
Lack of access to knowledge	12 (35%)	“We learned a lot of this stuff by mistake. A lot, a lot of stuff. That’s kind of why we end up being a little tight lipped. You know, with our information, that’s because a lot of this stuff’s been very, very expensive to come by.”
Lack of processing	10 (29%)	“...a big reason why this industry has not boomed because normally when you’re processing, you need a whole different license, it’s a lot of money, you need your own facility, you’d have spent like a quarter million dollars, a lot of farmers don’t have that.”
Lack of access to natural resources	8 (24%)	“And expansion, you know, every farm might not be able to expand, maybe because they don’t have enough water to support an expansion. Maybe they’re using all their water that they can use right now for their production now.”
Lack of fingerlings	8 (24%)	“...we were ready to put fingerlings in in the spring. But yeah, now we can’t get fingerlings. So we are going through our other tanks and we’re sorting them by size now to try and fill the RAS systems until we can get hopefully fingerlings in the fall.”
Pathogens/disease	7 (21%)	“...there’s a lot of pathogens, parasites, viruses, things like that, today, it seems like there needs to be more availability of trained individuals and pathologists to identify what these pathogens are, and be able to supply the direction and the corrective measures to control them.”
Lack of markets/marketing	6 (18%)	“I think the marketing is the next big stumbling block. A lot of people aren’t, they don’t have the market or they don’t see the bigger market. And that can be a major deterrent.”
Labor shortage	6 (18%)	“So there’s not a lot of people that say, Hey, I work on a fish farm, and I know about fish farming, and you can hire me and I can hit the ground running, that that labor base is not really there.”
Fish feed issues	6 (18%)	“Well, right now we have a major problem with feed. And I think it made it unprofitable for a lot of people.”
Predation	4 (12%)	“I know a guy who was doing hybrid bass which are done in cages, and he had an otter get in there and one otter basically wiped him out and he couldn’t afford to go back into business.”

#### Access to knowledge.

A sense of accessible knowledge contributed to higher PBC, with 26 of 34 producers (76%) feeling confident in their ability to find information through sources like universities, Extension programs, and peer networks. One producer noted the wealth of good information available online but stressed the importance of peer-to-peer and institutional contact: “Every new person getting into aquaculture should be talking to Extension. And they should also be talking to other farmers who are willing to talk that have been there.” Conversely, 12 participants (35%), often those with more years in the industry, recalled a lack of access to such resources when they started.

#### Access to financial resources.

A lack of access to financial resources was a dominant theme that lowered PBC, mentioned by 26 of 34 participants (76%). Producers identified aquaculture as a capital-intensive industry that lenders perceive as uniquely risky. One interviewee powerfully contrasted the lending experience in aquaculture with that of traditional farming:

“...if you’re a row crop farmer, and you walk into Farm Credit, or you walked into my local small town farm banks, and you tell them you farm 80 acres, they’ll pretty much close their eyes and hit the calculator a few times and say, this is what I’m willing to loan you to put out your crop. And you walk in there, and you say, okay, I want to raise fish. And I need $100,000 for expansion of the fish farm, they basically look at you and say how much land you own and how many cars you got? And do you have $200,000 to pay back the $100,000? That’s the difference. So, unequivocally, hands down, it’s funding that’s going to hold this thing back.”

This difficulty extends beyond loans to a lack of grant money, which producers believe flows primarily to academic research rather than to “the brick-and-mortar expansion that they need.” The few producers with high PBC in this area typically had unique financial situations, such as having saved up capital from other jobs or businesses to fund their farm.

#### Regulations.

Regulations were a prevalent and complex topic. While 24 producers (71%) felt the landscape was generally fair, 13 (38%) felt over-regulated. Experiences varied significantly based on location, species, and scale, but three common challenges emerged.

First, producers described a “one-size-fits-all” approach, where small farms face the same costly standards as large corporate operations. Required pathogen testing (e.g., for viral hemorrhagic septicemia, a viral fish disease) was a primary example, with its high costs acting as a significant barrier for smaller businesses. One producer argued the state should absorb the cost of mandated testing:

“I mean, my God, it’s a drop in the bucket to have 10 or 20 farms in the state tested, you know, we’re talking $20,000 to $30,000 to $40,000. I mean, they can blow that on a goddamn truck. A third of a truck. You know, they come up with $3 million grants and all kinds of stuff to build these incredible hatcheries. And the thing is, what would support the private guys a little bit, pay for our testing! Why do we have to pay for the testing? I mean, you’re requiring it, so why don’t you pay for it, you know?”

Second, some regulations deter growth. For instance, the National Pollutant Discharge Elimination System (NPDES) permit threshold of 9,090 harvest weight kilograms per year (approximately 20,000 pounds; [33]) incentivizes some farms to stay small to avoid an additional regulatory layer, even if it limits their profitability as a standalone business.

Third, a critical barrier was the lack of clear regulatory guidance. Producers expressed a desire to comply but struggled to navigate a non-standardized process where government agencies often lack aquaculture-specific knowledge. One interviewee captured the widespread frustration:

“I mean, there’s no paper out there that says you’re starting an aquaculture company, you need these six permits. I mean, it’s just not...you just go, and one government entity doesn’t know the other government entity. So, you call them, and you go, well, I need an aquaculture permit. ‘Okay, well, we can get that for you.’ And you ask them, was there any other permits? ‘Well, we don’t really know of any. This is just kind of our thing. Our part of it.’ And so, yeah, I called some veterinarians and the Board of Animal Health. I mean, they were able to help point me in some right directions, but they didn’t have definite answers. And it’s just, it’s not anyone’s fault. It’s just, there’s no, we’re not standardized, like cattle, chickens, and hogs.”

#### Support systems.

A pervasive theme lowering PBC was the feeling that aquaculture lacks the structural support afforded to other agricultural sectors. A major point of frustration was the absence of a “federal safety net” like crop insurance, which leaves producers to absorb all losses in a high-risk industry. One participant explained, “...if a blizzard goes through Montana...and they lose 200 head of cattle…there are programs to offset those losses. In our state, if somebody loses a bunch of fish, there are no programs. You eat the loss.”

This disparity was linked to a fundamental struggle for legitimacy; one producer recounted spending $50,000 in court “just to get aquaculture included in the definition of agriculture.” This lack of official recognition corresponds with a perceived lack of support from state agencies and a decline in vital university Extension programs. Further, producers cited a lack of accessible veterinary services for identifying and treating pathogens as a major operational hurdle that can lead to devastating financial losses.

Additionally, and possibly as a result, producers feel the aquaculture industry is not being advocated for or promoted in the same ways as other forms of agriculture, especially by the government and other important regulatory agencies. As one producer puts it,

“(Department of Natural Resources) policy toward aquaculture is important. It’s got to be positive. And I would like to see more support from Department of (Agriculture). Those are the two agencies that are everything to aquaculture. And we need to have positives from both sides.”

In addition to government programs (e.g., insurance, subsidies, loans, grants), many producers expressed feeling unsupported by the academic institutions in their state. This varied greatly between states that were more or less “aquaculture friendly,” but we saw a pattern in some states where Extension programs were lost or removed over time, despite producers expressing how useful and important it was to them. According to one producer, “It used to exist. It was fantastic. We lost it, oh, it’s probably been now almost 10 years. I mean, our industry was at the peak when we had Extension.” Lack of Extension understandably coincided with small or nonexistent aquaculture programs at the state universities nearby.

#### Social license.

Finally, the struggle for social license, or public acceptance, emerged as a barrier. Producers felt they were fighting against a negative public perception fueled by misinformation and “horror stories” about aquaculture. This required them to spend significant time and energy educating a skeptical public. One producer stated the challenge bluntly: “I think there has been a very conscientious and intentional effort by some to de-market domestic aquaculture products and to demonize domestic aquaculture production.” Overcoming this, participants felt, is essential for industry growth and requires a concerted effort in public education.

### Past behavior

While not part of the original TPB framework, we analyzed past behavior to understand how prior experiences with expansion or diversification influenced current intentions. We divided the themes regarding past behavior into three main categories: successful past experience, unsuccessful past experience, and no past experience (Table 4). Most producers (31 of 34; 91%) had direct experience in this area, with most of those new to the business being the only exceptions. These past experiences, whether successful or not, clearly shaped producers’ perspectives on future growth.

**Table 4:** Past behavior themes and example quotes.

Past Behavior		
Theme	# of interviews	Exemplary Quotes
**Level of past success**		
Successful	21 (62%)	“I think they were both both successful. Even after the initial--at the other farm--after the initial expansion, we sold those fish. We didn’t have a problem selling those fish. So that that was a success for me.”
Unsuccessful	6 (18%)	“And so I would have to be very careful to say, yes, it was the right thing to do and it was a great time to expand into other fish. But I probably would have been better off to hold off actually and continue raising stripers another two to three years and hold back a little bit. I think I would have been more successful financially.”
No past experience	3 (9%)	“No, we’re a new business. So we’re sitting where we want to be right now.”
**Types of past behavior**		
Physical expansion	20 (59%)	“...because we had all this empty space. And so we went from literally like a 300 gallons system, to two 2,000 gallon systems. And then after that, we added three more, and then four more after that.”
Species diversification	9 (26%)	“I mean, there is diversification already kind of in my history just from going from the hybrid striped bass to large mouth bass.”
Expansion of fish yields	6 (18%)	“...you know our growth in the past has been, the goal has been to increase the densities of what a tank can produce.”
Market diversification	6 (18%)	“Year round we provide to grocery stores and restaurants and farmers markets, we do a fresh, a frozen, a smoked, a smoked spread, and a pickled that we we do all on site.”
System diversification	5 (15%)	“...I diversified when he came out here and helped me build the system in my barn where I’m raising the fingerlings. And then I guess the other way I diversified is last year was the first year I put fingerlings in a cage to overwinter them.”

Producers with successful past expansions most often cited profitability and marketability as their key barometers of success. A project was deemed successful if it turned a profit and met persistent market demand. As one producer explained, they know their expansion was successful “because it’s profitable right now. I mean, it’s making money, it’s turning a profit. And we still can’t grow enough fish. So, demand is still there, and we haven’t been able to meet, to completely meet the demand.”

Furthermore, diversification was seen as a critical success factor for resilience. Several producers credited their varied market channels with saving their business during the COVID-19 pandemic, which disrupted key revenue streams like restaurant sales. One participant shared this experience:

“…especially with last year with COVID and even going into this year, our diversification as far as the business goes, helped us get through. Where we lost some of the agritourism side, we picked up on the weekends with people looking for things to do that were outside…so we’re able to weather some of those storms that other farmers that are maybe just producing fish for fresh sales just aren’t able to do, especially with a lot of the restaurants closing down last year.”

Conversely, unsuccessful past experiences were attributed to the same factors that lower perceived behavioral control, including disease outbreaks, regulatory barriers, and market instability.

A key theme emerging from past experiences was a deep skepticism toward rapid, large-scale, or technologically driven growth. Many respondents held a strong belief that slow, intentional expansion is the most successful model. They viewed large, high-investment expansions with distrust, citing a history of failures. As one industry veteran stated:

“Well, my business is successful. I’ve been here 50 years. I’ve seen a number of other facilities go in with millions of dollars invested with recirculating systems and all these other things. And not one single one has survived. I believe small farms are real farms. And family farms are the answer to aquaculture and not try to go big.”

This perspective extended to a critique of technologies often promoted as the future of the industry. Many producers expressed distrust in folks who are always pushing the ‘next big thing’ in aquaculture, and they tend to stick with what is tried and true and financially feasible for their operation:

“And, you know, I’ve been told for years, for a couple decades now, that the only way that aquaculture in the United States can hope to expand is to go to full recirculating aquaculture systems or aquaponic type systems, without having connection to water and conducting, you know, visible discharge. And that’s all fine and dandy, and it’s very entertaining. But the fact of the matter is that those systems fail economically, every time. And until those types of technologies actually become economically viable, it’s irrelevant to try to talk about them. And it’s disastrous for a business to engage in that type of an expansion.”

## Discussion

Our results show that the constructs of the Theory of Planned Behavior (TPB) allow us a rich, nuanced understanding of farmers’ intentions to expand (or not expand) their farms. Our study is situated in a long history of using TPB to understand farmer behavior. In the past, TPB has been used to explain small farmers’ intention to diversify their farms [25], adopt more sustainable farming practices [26,27], and other farm management practices. However, its application in fish farming has been limited. Here, we add to our understanding of how TPB can be used to analyze behavioral intentions while exploring key questions about aquaculture expansion in the US Great Lakes states.

However, TPB is not the only way to analyze behavioral intentions, and the TPB constructs (attitudes, norms, and perceived behavioral control) are not the only potential influences on future behavior. By adding past behaviors to the framework, we were able to understand key drivers of attitudes toward expansion: those who had been successful in the past were more willing to expand in the future. While this relationship between past success and future plans is self-evidently rational, the skepticism that farmers expressed about expanding too quickly or being too reliant on technology reveals that past experiences can influence not only *whether* to expand, but also *how* to do it. As suggested by past research [24], this context-specific addition to TPB enriches our understanding of the drivers of behaviors in the industry,

The widespread intention among aquaculture producers to expand or diversify stands in stark contrast to the industry’s relatively stagnant growth. The findings of this study suggest that the observed dissonance does not stem from limited ambition or lack of peer support (the latter of which was overwhelmingly favorable) but is instead rooted in substantial, intersecting barriers. The primary obstacles are low perceived behavioral control (PBC), compounded by specific unfavorable attitudes and negative past experiences that temper enthusiasm for growth.

The remainder of this discussion focuses on interpreting how these factors, particularly the formidable challenges related to PBC, create a landscape where intention often fails to translate into action.

### Barriers to expansion and diversification

#### Perceived behavioral control.

As the TPB framework suggests, desire is insufficient when the perceived ability to act is low. Our findings confirm that low perceived behavioral control (PBC) is the primary factor suppressing producers’ strong intentions to expand. This is not due to a single issue, but rather a complex web of interconnected structural barriers that producers feel are largely beyond their control. The most significant of these are access to capital, a dysfunctional regulatory environment, inadequate institutional support, and a challenging struggle for social license.

Access to capital is the foundational perceived barrier. Producers identified the capital-intensive nature of aquaculture, compounded by a systemic unwillingness from lenders to finance business expansion, as the most significant bottleneck holding back the industry. This lack of accessible capital creates a high-risk environment where all subsequent business decisions, from navigating regulations to absorbing losses, become more precarious. Prior work on oyster farming in Virginia has shown how capital structures and leasing issues are a significant barrier to aquaculture expansion [20]. Here, we show that mismatched policy mechanisms are a perceived problem for Great Lakes producers, as well. The regulatory landscape for aquaculture remains complex [34] and heterogeneous [21]. This complexity underscores the need for further research understanding how different policies influence aquaculture industry growth.

The regulatory environment further constrains producers, not merely through specific rules, but through a systemic lack of clarity, standardization, and scale-appropriate nuance. For many, the uncertainty created by poor regulatory guidance was a more significant barrier than the regulations themselves. This dysfunction manifests in two ways: “one-size-fits-all” rules, like costly pathogen testing, disproportionately burden small farms, while certain size-dependent regulations such as the NPDES permitting requirements inadvertently create a ceiling that discourages growth beyond a specific production threshold. In both cases, the regulatory structure actively works against expansion. Aquaculture policy differs considerably across states, making it difficult to establish causal links between specific policies and industry growth [21]. Nonetheless, the negative perception of the policy environment expressed by our interviewees appears to hinder aquaculture business expansion.

This precarious position is exacerbated by a perceived lack of institutional support, especially when compared to other agricultural sectors. Producers identified the absence of safety net programs, like crop insurance, as a critical vulnerability that amplifies the industry’s inherent risks. This institutional neglect extends to a lack of accessible veterinary services and a decline in state-level Extension support, leaving producers feeling isolated in key areas of need, particularly in managing disease outbreaks. The slow and cumbersome process for pathogen identification, for example, directly translates to financial loss, making the risk of expansion feel unmanageable.

Finally, producers perceive a lack of social license, or public acceptance, which lowers their perceived control over their own success. Social license theory is fairly well developed but under-tested, particularly in aquaculture [35]. While our findings don’t test the theory directly, they do underscore the need for more research on social license as the producers report feeling embattled by a public perception shaped by what they perceive as misinformation. This external struggle, combined with the internal structural barriers, creates a formidable set of challenges that systematically suppress the desire for business expansion.

#### Attitudes toward the behavior.

While producers’ attitudes were mixed, they reveal a core conflict between the intrinsic, mission-driven rewards of aquaculture and the often-unfavorable realities of its business environment. The favorable attitudes, including pride in producing local food, meeting consumer demand, and contributing to sustainability, are the key factors that keep producers in the business. However, these positive feelings are counterbalanced by a set of pragmatic, unfavorable attitudes that temper intention to expand.

The most powerful of these unfavorable attitudes is the frustration with low profitability, particularly in the food fish sector. This is not merely a preference but a calculated business reality that often cannot justify the significant investment required for expansion. This financial reality is inseparable from the other key unfavorable attitudes: the perception of the industry as capital-intensive and high-risk, and the concern over the excessive workloads that expansion entails.

Importantly, these attitudes do not exist in a vacuum; they are shaped and validated by the structural barriers identified above. The unfavorable attitude toward financial risk, for example, is a rational response to the lack of access to capital and institutional safety nets. Similarly, concerns over excessive workloads are magnified by the difficulties in finding and affording skilled labor. Therefore, while producers hold a favorable vision for their industry’s potential, their attitudes toward the specific behavior of expansion are dominated by pragmatic concerns that are constantly reinforced by their external environment. Prior research (e.g., [28]’s work on pesticide use) has shown an interaction between attitudes and perceived or actual behavioral control. Our study affirms this while adding to our understanding of how attitudes interact with diverse and daunting perceived and actual barriers to performing intended behaviors.

#### Past behavior.

Prior quantitative research (e.g., [24]) has shown that incorporating previous behaviors into models can increase the models’ predictive power. This qualitative study shows how this relationship can be multifaceted, with past behavior influencing not only the desire to expand, but how producers might choose to expand. Producers’ past experiences introduce a crucial tension that shapes their approach to future growth. On one hand, direct, personal successes with expansion or diversification, such as selling out of a new product or weathering the COVID-19 pandemic due to diverse market channels, reinforce a positive attitude toward the concept of growth. On the other hand, this optimism is heavily tempered by a powerful, collectively held narrative about the failure of large-scale aquaculture ventures. Producers draw from their own observations and industry lore to form a cautionary belief that “bigger isn’t always better.” The specter of high-investment, high-tech operations that have consistently failed serves as a powerful deterrent. The resulting mindset favors slow, intentional, and risk-averse growth over ambitious expansion. This interpretation aligns with the PBC barriers discussed previously. When capital is scarce and the regulatory environment is uncertain, a “slow and steady” approach informed by past industry failures becomes the most rational path forward.

#### Subjective norms.

Subjective norms can strongly influence farmers’ decisions to diversify their operations [25] and adopt different conservation practices [26–28]. Subjective norms were strongly favorable across the board in our interviews. Potentially because of the small size of the food fish aquaculture industry in the region, there appeared to be very low levels of competition amongst producers. Many expressed feeling as though their peers want them to succeed and felt no pressure from them not to expand or diversify their business. In fact, many felt that their peers would feel positively about seeing any expansion in the industry, as a means by which “a rising tide lifts all boats.” It seemed to be a commonly held notion that that the more success that is seen in the industry would likely benefit everyone, whether it be from changing perceptions of consumers and lenders or increasing the affordability and availability of equipment needed for business operation. These norms are likely sufficient to encourage expansion or diversification: the region’s slow growth is likely due to other factors.

### Limitations

This study had several limitations. First, as a qualitative study, our findings are intended to provide theoretical depth rather than statistical generalizability. We utilized purposive sampling through our professional networks, which was essential for accessing this specialized population, but this approach means our sample may not be fully representative of all producers in the Great Lakes region, particularly those not connected to existing industry or university networks. Furthermore, while we have been transparent about our researcher characteristics, our positionality as researchers affiliated with a university and a Sea Grant-funded aquaculture collaborative inevitably informed our interpretations and may have influenced how participants responded to questions regarding institutional and academic support.

Second, our study is subject to the “intention-behavior gap” that is a known feature of the Theory of Planned Behavior, where stated intentions do not always translate into actual behavior for various reasons. This is compounded by the study’s temporal context; data were collected in 2021 when the economic disruptions of the COVID-19 pandemic were particularly salient for producers. The intentions captured here represent a specific moment in time and may have since been altered by subsequent economic shifts or other factors not captured in this study.

Finally, our analysis relies entirely on self-reported data from semi-structured interviews. While this method is ideal for capturing the rich, nuanced perspectives that were the focus of this research, it is subject to potential recall and social desirability biases. The accounts of past business successes or failures and the stated intentions for future growth are filtered through each producer’s personal memory and subjective framing. Therefore, the findings should be understood as representing the producers’ valuable perceptions and experiences, rather than an audited, objective record of their business operations.

### Conclusions

Despite the industry stagnation, most producers expressed strong intentions to expand or diversify their operations. From the producers’ perspective, the lack of growth in the industry is not driven by a lack of ambition or peer support, but by structural barriers that hinder producers’ ability to expand or diversify their businesses. Producers feel constrained by a financial system that deems them uniquely risky, a regulatory environment that lacks clarity and discourages scaling, and an absence of the institutional support systems, like risk management programs and Extension services, that are foundational to other agricultural sectors.

Access to capital is a formidable constraint. Aquaculture’s capital-intensive nature, combined with lenders’ perception of the industry as high-risk, creates a financial bottleneck that limits producers’ ability to act on their intentions. This lack of accessible financing amplifies other risks, making expansion feel untenable even for highly motivated producers.

Second, regulatory complexity and uncertainty disincentivize growth. Producers face a fragmented, non-standardized permitting process and “one-size-fits-all” rules that disproportionately burden small farms. Size-based thresholds, such as those in NPDES permitting, unintentionally create ceilings that discourage scaling beyond certain production levels. These regulatory dynamics not only increase costs but also erode confidence in the feasibility of expansion.

Third, institutional neglect compounds these challenges. Unlike other agricultural sectors, aquaculture lacks safety nets such as crop insurance, leaving producers vulnerable to catastrophic losses. The lack of broadly available Extension programs and limited veterinary services further isolate producers, particularly in managing disease outbreaks—a risk that becomes more acute with expansion.

Finally, producers struggle for social license. The producers perceive negative public attitudes toward aquaculture, forcing farmers to invest time and resources in education and outreach, diverting attention from growth. This external pressure reinforces the internal constraints identified above.

Overall, these perceived barriers are a substantial hinderance to expansion plans. If the industry is going to grow, these perceived risks need to be alleviated to some degree. As many producers expressed in our interviews, change is needed to make this industry more lucrative, successful, attractive and accessible to newcomers.
